# A shared frailty model for assessing time to seizure remission in adults with epilepsy

**DOI:** 10.1038/s41598-025-17991-2

**Published:** 2025-09-01

**Authors:** Abiy Disasa Jote, Mesfin Esayas Lelisho, Wegayehu Enbeyle Sheferaw

**Affiliations:** 1 Department of Statistics, College of Natural Science, Jimma University, Jimma, Ethiopia; 2 Department of Statistics, College of Natural and Computational Sciences, Mizan-Tepi University, Tepi, Ethiopia; 3 Institute of Health Research, Faculty of Health Science, University of Canberra, Bruce, Australia

**Keywords:** Epilepsy, Seizure remission, Survival analysis, Shared frailty, Clustering effect, Epidemiology, Public health

## Abstract

Epilepsy remains a significant global health concern with increasing prevalence and incidence. This study aimed to model the time to first remission among epilepsy patients at Jimma University Medical Center, Ethiopia, using parametric shared frailty models. A retrospective study was conducted on epilepsy patients treated between 1st January 2018 and 30th December 2023. All patients received anti-seizure medications (ASMs) upon enrollment. Additionally, 12% of the cohort had received ASM treatment prior to enrollment outside JUMC. Log-logistic, log-normal, and Weibull baseline hazard functions were combined with gamma and inverse Gaussian frailty distributions to model time to first remission. Model selection was based on the Akaike Information Criterion (AIC). The median time for patients to achieve their first seizure remission was 38 months, with 45.5% (95% CI: 40.9–50.1%) of patients experiencing remission. The variability in remission times across different districts, as modelled by the log-normal-inverse Gaussian shared frailty model, was estimated to be θ = 0.454. Patients aged 25–44 years (acceleration factor 1.13 [95% CI 1.04–1.23], *p* = 0.005], those with more than five pre-treatment seizures (acceleration factor 1.08 [95% CI 1.02–1.15, *p* = 0.018]), and individuals with focal epilepsy (acceleration factor 1.15 [95% CI 1.07–1.25, *p* = 0.003]) were associated with significantly longer remission times compared to other patient groups while those with good treatment adherence (acceleration factor 0.88 [95% CI 0.81–0.96, *p* = 0.005]) were associated with significantly shorter remission times compared to poor treatment adherence. The log-normal-inverse Gaussian shared frailty model offers valuable insights into the variability of remission patterns among patients. Specifically, individuals aged 25–44 years, those with a history of more than five pre-treatment seizures, and patients with focal epilepsy experienced significantly longer remission times. In contrast, patients who adhered well to their treatment regimens achieved remission more quickly than other groups.

**Supplementary Information:**

The online version contains supplementary material available at 10.1038/s41598-025-17991-2.

##  Introduction

Epilepsy is a neurological disorder characterized by a chronic predisposition to recurrent epileptic seizures, which can lead to a variety of neurobiological, psychological, and social consequences^1,2^. Epilepsy is diagnosed after two unprovoked seizures ≥ 24 h apart or one seizure with ≥ 60% risk of recurrence^3^. The clinical manifestations of seizures can vary widely, depending on the brain region where the disruption begins and how far it spreads^4^.

Globally, epilepsy represents a significant health burden, affecting approximately 50 million individuals^5^. Each year, an estimated five million people are newly diagnosed with epilepsy^6^. Alarmingly, of the estimated 50 million people living with epilepsy worldwide, approximately 75% in low-income countries and over 50% in middle-income countries do not receive adequate treatment, despite evidence that 70% of individuals could achieve seizure freedom with appropriate medical care^7^. While the global treatment gap varies significantly by region, sub-Saharan Africa faces some of the highest disparities, with gaps exceeding 75–90% in certain areas^8,9^. In high-income countries, this gap drops to less than 10%, reflecting stark inequities in healthcare access^7,9^. Of the majority of those living with epilepsy, nearly 80% reside in low and middle-income countries^10^. In Africa alone, approximately ten million people are affected, particularly among children, adolescents, and the elderly^11^. In sub-Saharan Africa, active epilepsy is estimated to impact four million individuals, with a notably high prevalence^12–15^. Despite advancements in diagnosis and treatment, about eight million people in Africa remain untreated with modern antiepileptic drugs due to various barriers, including financial constraints, lack of awareness regarding the treatability of epilepsy, and limited access to healthcare facilities^16^. In Ethiopia, the prevalence of epilepsy is reported to range from 5.2 to 29.5 per 1,000 individuals^17,18^.

Several factors can influence the likelihood of achieving remission in epilepsy. These include the type of epilepsy and the frequency of seizures; for instance, patients with focal epilepsy often experience better remission rates compared to those with generalized epilepsy^19^. Early diagnosis and treatment, along with adherence to prescribed medications, significantly enhance the likelihood of achieving remission^20,21^. Additionally, demographic factors such as age where younger patients generally have more favorable outcomes^22^. Comorbidities including intellectual disabilities and psychiatric disorders can hinder remission, while lifestyle factors like sleep hygiene and stress management also play a role^23,24^. Genetic predispositions, initial treatment responses, and the choice of antiepileptic drugs further impact outcomes, with seizure freedom within the first year being a strong predictor of long-term remission^25–27^. Finally, psychosocial factors, including social support and healthcare access, are vital for optimizing treatment and improving remission rates^28,29^.

To evaluate the various factors associated with remission in epilepsy, survival analysis can be employed to predict and statistically estimate the time to remission^30^. Survival analysis is a statistical method used to analyze data that measures the time until a specific event occurs^31,32^. This study aimed to model the time to first remission of epilepsy among patients at Jimma University Medical Center (JUMC) using parametric shared frailty models. Weibull, log-logistic, and lognormal baseline distributions, along with gamma and inverse Gaussian frailty distributions, can be applied to evaluate the determinants affecting the time to first remission in epilepsy patients.

## Methodology

### Description of data source and study area

This study was based on epilepsy patients under follow-up at JUMC, from January 1, 2018, up to December 30, 2023. The data utilized was secondary, derived from hospital records spanning from the date of admission to either the date of remission or when patients were censored.

### Study design

A retrospective cohort study was conducted involving epilepsy patients who visited JUMC over five years.

### Study population

Patients included in this study had a clinical diagnosis of epilepsy confirmed by a neurologist based on the International League Against Epilepsy (ILAE) criteria, defined as having experienced two or more unprovoked seizures occurring at least 24 h apart, or one seizure with a high risk (≥ 60%) of recurrence^3^. Only patients meeting these criteria were considered epilepsy cases eligible for this study.

Participants attended clinic follow-ups monthly, during which seizure status and medication adherence were assessed. Access to antiseizure medications was primarily through the clinic’s pharmacy, with some participants obtaining medications from local health centers. Adherence to medication was evaluated both through direct patient interviews during these visits and by using the Medication Adherence Rating Scale (MARS). Telephone follow-ups were conducted as needed to reinforce adherence and monitor any adverse events. The study population comprised new epilepsy patients who received follow-up at the same clinic from January 1, 2018, to December 30, 2023. Patient documents were reviewed until December 30, 2023.

### Inclusion-exclusion criteria

**Fig. 1:**
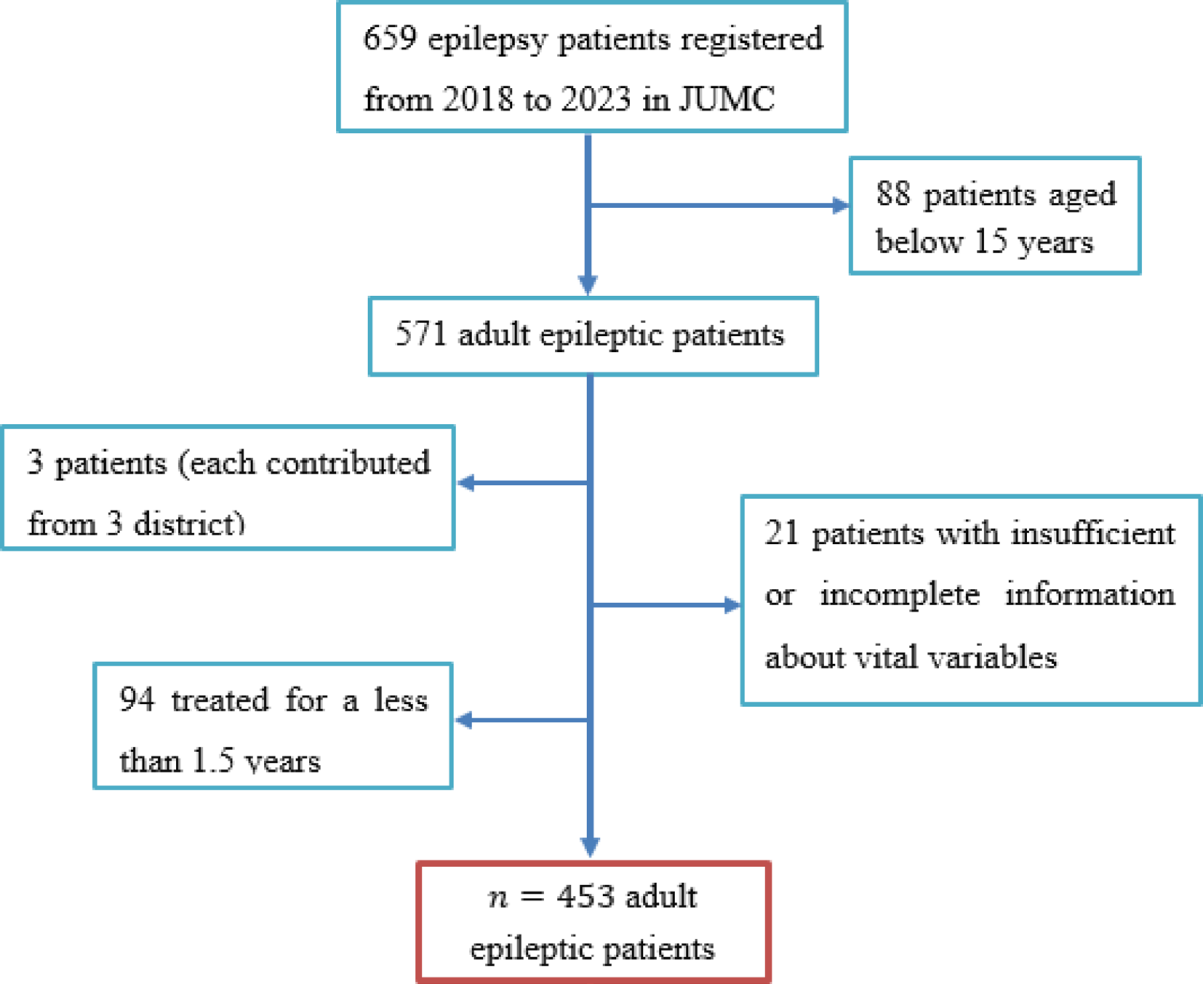
Conceptual framework of sampling patients’.

In this study, we included epilepsy patients over the age of 15 with sufficient information about vital variables who were under follow-up at JUMC. This age group was chosen because epilepsy tends to have a more significant economic impact in terms of lost work productivity among adults than in the pediatric population. Additionally, patients were required to have received treatment for a minimum of one and a half years, as patients typically begin to experience seizure freedom after around six months of treatment initiation^20,21^. However, patients with incomplete records regarding vital variables and districts contributing only a single patient were excluded, as the shared frailty model requires at least two patients in a cluster (Fig. 1).

### Study variables

#### Dependent variable

- The dependent variable is the time to first remission, defined as the duration (in months) until the first twelve-month seizure-free period following the initiation of treatment in epilepsy patients. The event of interest is remission; otherwise, it is considered censored. Epilepsy patients who do not achieve remission during the study period or who are lost to follow-up before reaching remission are classified as censored.
- The 12-month seizure remission period was chosen based on its widespread acceptance as a clinically meaningful endpoint in epilepsy research. This definition aligns with the International League Against Epilepsy (ILAE) recommendations and is commonly employed in clinical and epidemiological studies to assess treatment outcomes^3,33^.

#### Independent variables

- Socio-demographics: gender, age at the diagnosis, and family history of epilepsy.
- Disease and medication-related variables: Seizure type, total number of pre-treatment seizures, pre-treatment duration, comorbidity, neurologic examination, adherence to the medication, and adverse effects.

#### Operational definitions

- Seizure remission: Defined as being free from all types of seizures for at least one year at any time during the follow-up period. This includes early remission, which is achieved within the first 6 months, and late remission, defined as achieving remission more than 6 months after the initiation of treatment.
- No remission: Refers to the inability to attain seizure remission throughout the entire follow-up period.
- Pre-treatment duration: The time span from the onset of seizures to the start of anti-seizure medications (ASMs). For this study, we operationalize it as the time elapsed from seizure onset to the participant’s initial presentation at our clinic, irrespective of any prior ASM therapy received elsewhere.
- Adherence to ASMs: Adherence to anti-seizure medications (ASMs) refers to the extent to which patients follow their prescribed treatment regimen^34^. Medication adherence was evaluated using the ten-item Medication Adherence Rating Scale (MARS), a validated tool originally developed for psychosis and subsequently validated in diverse patient populations including those in Nigeria^35^. The scale has been adapted for use in epilepsy patients to measure adherence, with scores ≥ 7 indicating good adherence.

### Data quality control

To ensure data quality, the data collection tool underwent pre-testing, data collectors were trained, and supervision was maintained throughout the data collection process. The instrument was initially tested on 5% of the sample, resulting in necessary adjustments. Data collectors were trained on the data collection format and effective recording methods. Throughout the data collection period, investigators closely supervised the process by reviewing completed data forms and offering feedback to the data collectors. Continuous close supervision by investigators who reviewed completed data forms regularly, provided timely feedback to data collectors, and corrected data entry errors. Addressing missing or inconsistent data by cross-checking with source documents and, where feasible, verifying information via follow-up calls.

### Method for statistical analysis

We applied the Kaplan-Meier estimator to estimate the survival function, accounting for censored data. A parametric shared frailty model was employed to assess the impact of covariates on survival times while accounting for unobserved heterogeneity among subjects, with frailty distributions specified as gamma or inverse Gaussian. Furthermore, the frailty model introduced by Vaupel can be applied^33,34,36,37^ and further discussed in the literature, accounts for heterogeneity in baseline characteristics.

A univariable parametric shared frailty model was conducted to assess the relationship between each independent variable and seizure remission. Following this, variables that demonstrated a p-value of ≤ 0.25 in the univariate analysis were incorporated into a multivariable parametric shared frailty model to pinpoint independent predictors of seizure remission.

The effect of covariates was expressed as acceleration factors (AFs), which indicate the relative change in time to event. An acceleration factor greater than 1 signifies an extended time to seizure remission, indicating that patients achieve remission later compared to the reference category for the same covariates. Conversely, an acceleration factor less than 1 suggests earlier remission relative to the reference. The 95% confidence interval (CI) was used to assess the precision of the AF estimates; significance is indicated if the 95% CI does not include 1.00, which represents no effect.

Model comparison was conducted using the Akaike Information Criterion (AIC) and likelihood ratio tests to identify the best-fitting model. Additionally, Kendall’s Tau^38,39^ was utilized to evaluate associations in survival times, with corresponding p-values to assess the strength of these associations.

## Results

### Summary of patients’ characteristics diagnosed with epilepsy

#### Socio-demographic characteristics

A total of 453 adult epilepsy patients from 18 districts participated in the study, with a slightly higher proportion of males (55.4%) than females (44.6%). The overall remission rate was 45.5%, with males showing a marginally higher remission rate (45.82%) compared to females (45.05%) though this difference was not statistically significant (*p* = 0.65). Age significantly influenced remission outcomes: the 15–24 year age group had the highest remission rate at 55.24% and the shortest median time to remission of 34 months, while the 25–44 year group had a lower remission rate of 34.9% and experienced a longer time to remission (*p* = 0.008). Older patients aged 65 and above had the longest median duration to remission at 41 months. These findings suggest that younger adults tend to achieve remission more quickly and at higher rates than older patients, highlighting the need to consider age-related factors in managing epilepsy (Table 1).

## Clinical and disease-related characteristics

Analysis of clinical characteristics revealed that patients with GCTS comprised 82.8% of the sample, achieving a remission rate of 48.3%. In contrast, those with Focal Seizures (FCS) had a lower remission rate of 30.77%. The number of seizures prior to diagnosis significantly influenced remission outcomes; patients experiencing ≤ 5 seizures had a remission rate of 57.58%, while those with more than 5 seizures had a rate of only 36.60%. Additionally, a pre-treatment duration of ≤ 12 months was associated with a higher remission rate of 50.55%, further emphasizing the importance of early intervention.

Other characteristics included medication adherence, where good adherence resulted in a remission rate of 56.27%, compared to just 21.83% for poor adherence. Additionally, patients who did not report adverse effects had a remission rate of 45.72%. Neurological examinations revealed that patients with normal results experienced a higher remission rate of 46.4% compared to 34.28% for those with abnormalities. Comorbid conditions were present in 21.6% of cases, with a remission rate of 43.88% among those affected. Overall, the median time to remission in this study was 38 months (IQR: 36–41 months) (Table 1).

**Table 1:** Descriptive summaries of patient’s characteristics diagnosed for Epilepsy.

Covariates	Categories	*Status*		Total	Median (month)	IQR	95%CI	P-value (Chi-square)
		Censored N (%)	Remission N (%)					
Gender	Female	111 (54.95)	91 (45.05)	202 (44.6)	38	(36, 41)	(34,43)	0.65
	Male	136 (54.18)	115 (45.82)	251(55.4)	38	(36, 39)	(36,40)	
Age (in years)	15–24	81(44.76)	100(55.24)	181(40)	34	(33, 36)	(32,37)	0.008
	25–44	97(65.1)	52(34.9)	149(32.9)	40	(38, 42)	(36,43)	
	45–64	48(62.34)	29(37.66)	77(17)	40	(38, 42)	(37,43)	
	$\:\ge\:$65	21(45.65)	25(54.35)	46(10.2)	41	(40, 42)	(40,43)	
Seizure type	GCTS	193(51.47)	182(48.53)	375(82.8)	37	(35, 39)	(34,39)	0.003
	FCS	54(69.23)	24(30.77)	78(17.2)	42	(41, 44)	(40,45)	
Number of seizure	$\:\le\:$5	84(42.42)	114(57.58)	198(43.7)	35	(33, 37)	(32,38)	0.018
	$\:>$5	163(64.0)	92(36.0)	255(56.3)	41	(39, 42)	(38,42)	
Pre-treatment duration (in months)	$\:\le\:$12	136(49.45)	139(50.55)	275(60.7)	37	(35, 39)	(34,40)	0.24
	$\:>$12	111(62.36)	67(37.64)	178(39.3)	38	(36, 41)	(35,42)	
Family history	No	209(55)	171(45)	380(83.9)	38	(36, 40)	(36,41)	0.58
	Yes	38(52.05)	35(47.95)	73(16.1)	36	(34, 39)	(34,42)	
Adherence to medication	Poor	111(79.17)	31(21.83)	142(31.3)	43	(41, 45)	(40,47)	0.0003
	Good	136(43.73)	175(56.27)	311(68.7)	37	(35, 38)	(34,39)	
Adverse effect	No	165(54.28	139(45.72)	304(67.1)	38	(36, 40)	(36,41)	0.87
	Yes	82(55.04)	67(44.96)	149(32.9)	38	(35, 40)	(34,41)	
Neurological examination	Abnormal	23(65.72)	12(34.28)	35(7.7)	38	(36, 39)	(36,40)	0.15
	Normal	224(53.6)	194(46.4)	418(92.3)	38	(36, 39)	(35,40)	
Comorbidity	No	192(54.1)	163(45.9)	355(78.4)	36	(35, 38)	(34,39)	0.09
	Yes	55(56.12)	43(43.88)	98(21.6)	41	(40, 41)	(40,42)	

## Results of univariable analysis

Univariable analysis identified covariates that were statistically significant based on a 25% cutoff and eligible for multivariable analysis. These includes age group, seizure type, number of seizures prior to initial diagnosis, pre-treatment duration, date of diagnosis, adherence status, and comorbidity.

## Model comparison

Lognormal-Inverse Gaussian shared frailty model provided the best fit, as indicated by the lowest AIC value of 1686.94 (Table 2). The other parametric frailty models (Weibull, Lognormal, and Log-logistic with both Inverse Gaussian and Gamma frailty distributions) generally yielded similar covariate effects in terms of direction and significance, with minor variations in magnitude. Detailed results from these models are provided in the supplementary material (Supplementary Tables S1–S5), supporting the robustness of our findings.

**Table 2:** AIC values of the parametric frailty models.

Model		AIC
Baseline hazard function\	Frailty distribution	
Weibull	Inverse Gaussian	1706.55
	Gamma	1711.86
Lognormal	Inverse Gaussian	1686.94
	Gamma	1695.79
Log-logistic	Inverse Gaussian	1699.15
	Gamma	1700.53

AIC = Akaike’s information criteria

Analysis using the Lognormal-Inverse-Gaussian shared frailty model revealed that age group, seizure type, number of seizures prior to initial diagnosis, and adherence status were significant at the 5% level (Table 3). In contrast, pre-treatment duration and comorbidity did not reach significance.

An acceleration factor greater than 1 indicates an extended time to the first remission, while a factor less than 1 suggests that patients achieved remission earlier compared to the reference category for the same covariates. Specifically, patients aged 25 to 44 experienced a significantly longer time to first remission, with an acceleration factor of 1.13 [ϕ = 1.13, 95% CI: 1.05–1.27] compared to those aged 15 to 24. Similarly, patients with focal seizures had a prolonged time to first remission by a factor of 1.15 [ϕ = 1.15, 95% CI: 1.05–1.27] when compared to those with GCTS. The number of seizures before diagnosis was another significant covariate; patients with more than five episodes prior to diagnosis had an acceleration factor of 1.08 [ϕ = 1.08, 95% CI: 1.01–1.16], indicating a longer time to remission compared to those with five or fewer episodes. In contrast, patients who adhered well to their treatment achieved remission earlier, with an acceleration factor of 0.88 [ϕ = 0.88, 95% CI: 0.81–0.96] compared to those with poor adherence.

The shape parameter of the Lognormal-Inverse-Gaussian shared frailty model was 3.762, indicating an unimodal hazard function that increases to a peak and then decreases. The estimated variability (heterogeneity) within the population of clusters (woredas) was 0.454, significant at the 5% level, with approximately 18.5% (Kendall’s tau=0.185) of the dependence observed within clusters (Table 3).

**Table 3:** Multivariable analysis using the log-normal-inverse Gaussian frailty model.

Covariates	Categories	Coefficient	S.E.	ϕ	95%CI	*p*-value
Intercept		3.5104	0.0506	33.46	[30.3, 36.95]	
Age	15–24(ref)			1.00		
	25–44	0.1218	0.0431	1.13	[1.04, 1.23]	0.005*
	45–64	0.0578	0.0529	1.06	[0.95, 1.18]	0.270
	$\:\ge\:$65	0.0963	0.0641	1.10	[0.97, 1.25]	0.130
Seizure type	GCTS(ref)			1.00		
	FCS	0.1444	0.0488	1.15	[1.05, 1.27]	0.003*
Number of seizures before diagnosis	$\:\le\:$5(ref)			1.00		
	$\:>$5	0.0820	0.0345	1.08	[1.01, 1.16]	0.018*
Pre-treatment duration	$\:\le\:$12(ref)			1.00		
	$\:>$12	0.0427	0.0366	1.04	[0.97, 1.12]	0.240
Adherence	Poor(ref)			1.00		
	Good	-0.1216	0.0435	0.88	[0.81, 0.96]	0.005*
Comorbidity	No(ref)			1.00		
	Yes	0.0826	0.0479	1.09	[0.98, 1.19]	0.085
$$\:\:\:\:\:\varvec{\theta\:}=0.454\varvec{*}\:\:\:\:\:\:\:\:\:\:\:\:\:\:\:\:\varvec{\tau\:}=\:0.185\:\:\:\:\:\:\:\:\:\:\:\:\:\:\:\:\:\:\:\:\:\:\varvec{\rho\:}=\:\:\:3.762\:\:\:\:\:\:\:\:\:\:\:\:\:\:\:\:\:\:\:\:\:\:\:\:\:\:\:\varvec{A}\varvec{I}\varvec{C}=\:\:\:1686.94$$						

*p < 0.05 was statistically significant, ϕ = Acceleration factor, $\:\varvec{\theta\:}$ =Variance of the random effect, $\:\tau\:\:$=Kendall’s tau, AIC = Akaike’s Information Criteria, CI = confidence interval, S.E = standard error, Ref = Reference, $\:\rho\:$=shape parameter.

## Cox-snell residuals analysis

**Fig. 2:**
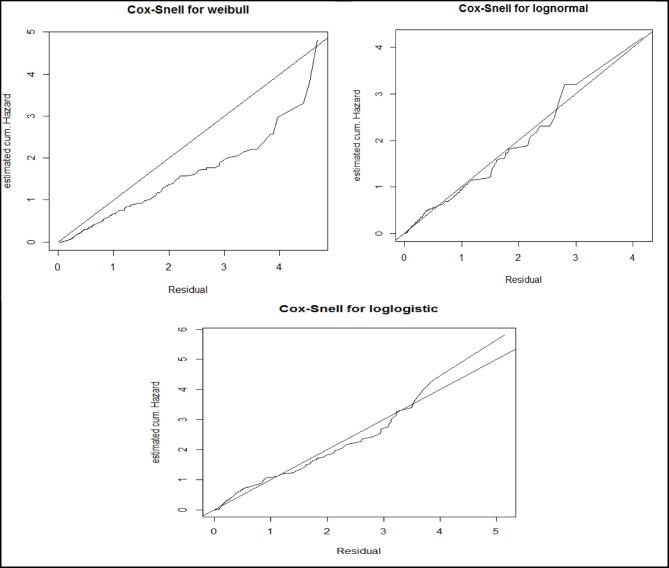
Cox-snell residuals plots of Weibull, lognormal and log-logistic models.

The Cox-Snell residuals plot reveals that the line corresponding to the log-normal models closely aligns with the diagonal line through the origin (Fig. 2). This proximity suggests that the log-normal model provides a superior fit for the epilepsy remission dataset compared to other models evaluated. Such findings underscore the robustness of the log-normal model in capturing the nuances of the data distribution. Additionally, this conclusion is supported by the earlier analysis presented in (Table 2), which utilized Akaike’s Information Criterion (AIC) for model comparison. The AIC measures the goodness of fit while penalizing for model complexity, and the log-normal model’s favourable AIC value reinforces its status as the preferred choice.

## Diagnostic plots of the parametric baselines

**Fig. 3:**
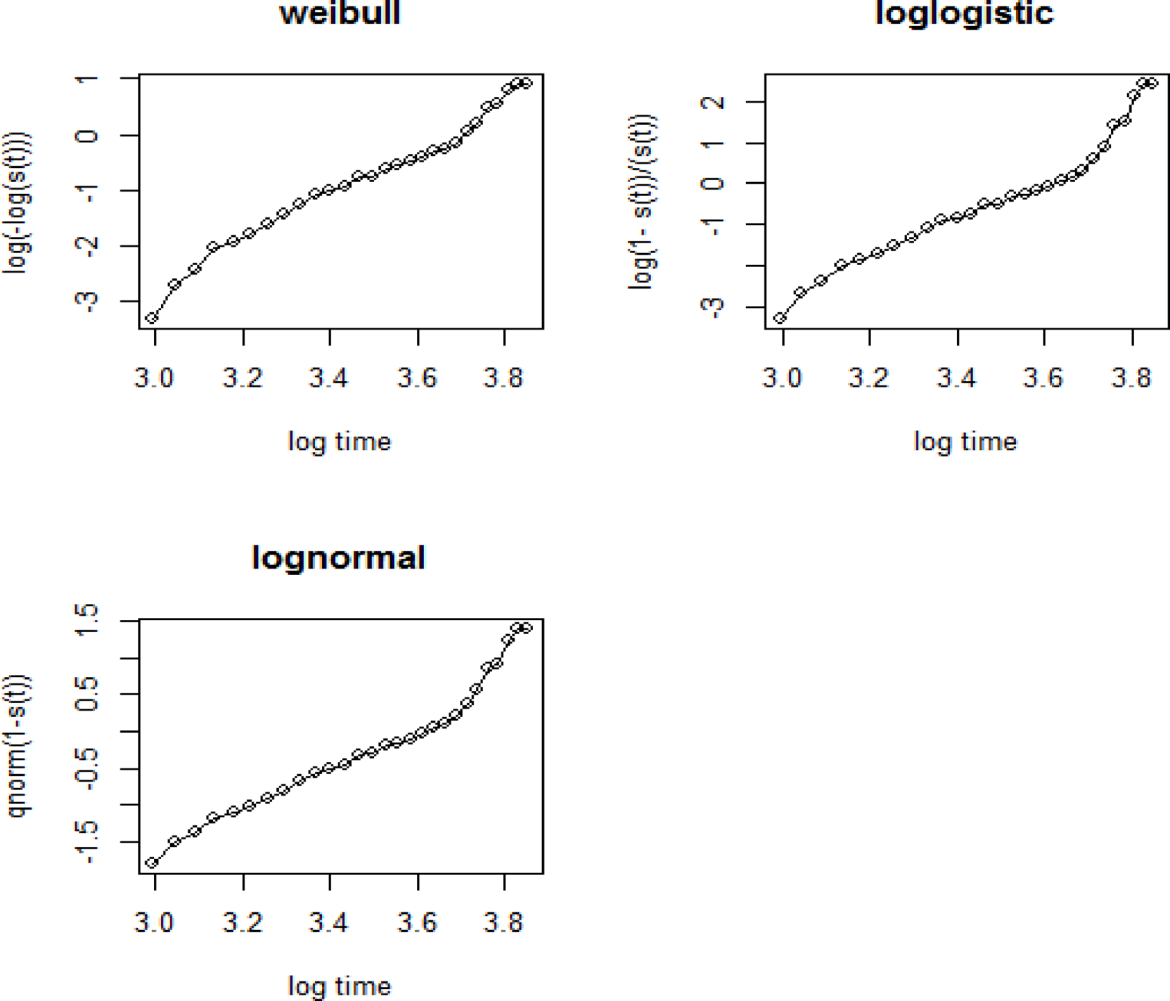
Graphical evaluation of the Weibull, log-logistic and log-normal assumptions.

The log-normal baseline distribution exhibits a more linear trend compared to both Weibull and log-logistic baseline distributions. This observation further supports the AIC-based selection of the log-normal baseline distribution as the most suitable for the given dataset (Fig. 3).

## Survival of epilepsy patients by different groups

**Fig. 4:**
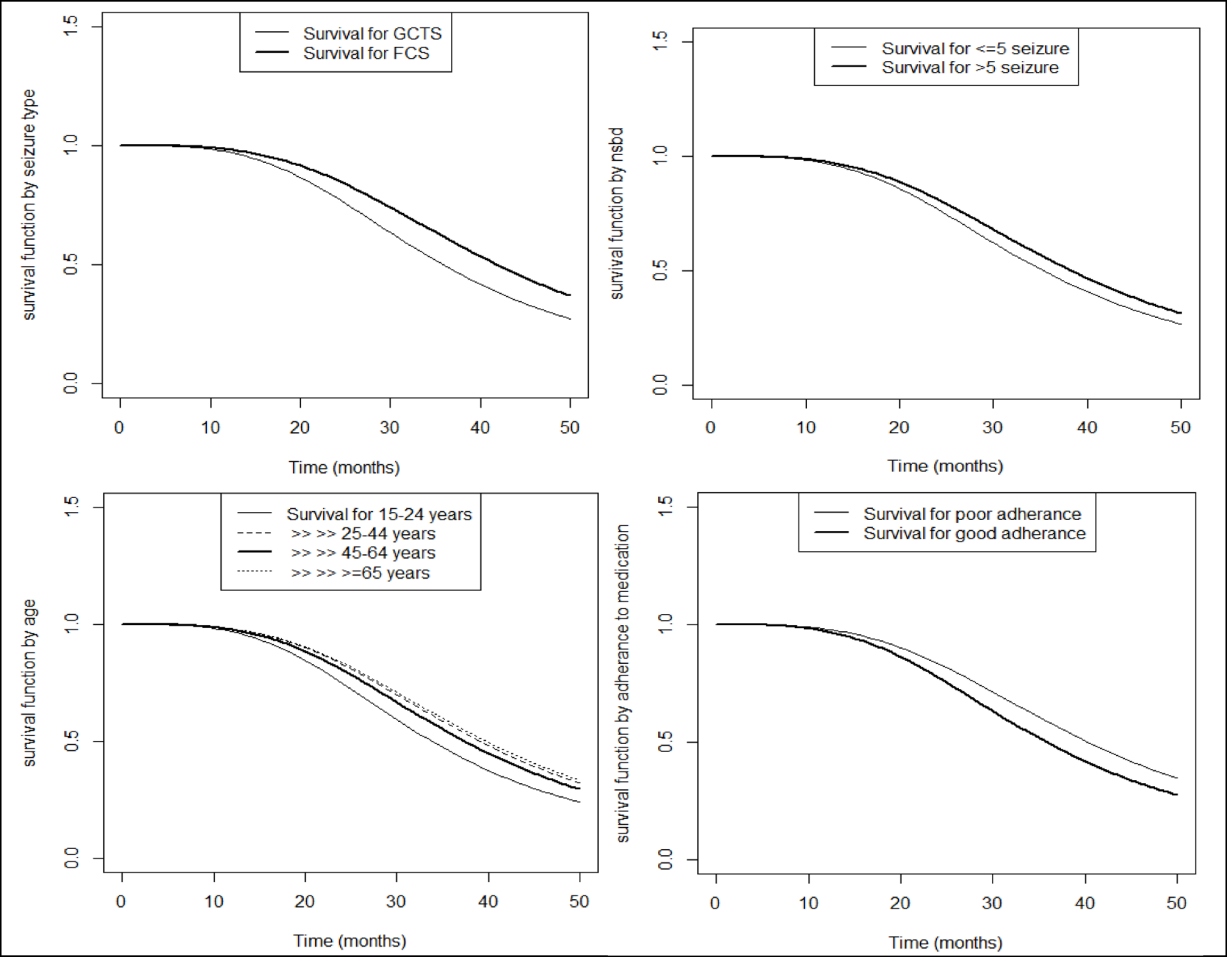
Survival functions by key covariates estimated from the log-normal-inverse Gaussian frailty model, illustrating differences in time to seizure remission across groups.

Figure 4 displays survival functions stratified by major covariates from the final log-normal-inverse Gaussian frailty model, complementing the numerical estimates reported in Table 3. The figure visually demonstrates differences in remission timing across groups, while Table 3 quantifies these effects with acceleration factors and confidence intervals.

Patients with Focal Seizures (FC) exhibit extended remission time than those with GCTS. Additionally, the probability of remission at a specific point in time is higher for patients aged 25–44 years compared to those in the 15–24 age group. Furthermore, patients with good adherence to treatment demonstrate faster remission than those with poor adherence. Lastly, individuals who had experienced more than five seizure episodes by the time of diagnosis tended to have a longer time to achieve remission compared to those with fewer than five episodes (Fig. 4).

## Discussion

This study focused on the positive outcome of achieving seizure remission in patients with epilepsy, revealing that only 206 (45.5%) patients with 95% CI: 40.9–50.1% attained remission. This finding aligns with previous research, which has reported varying remission rates in epilepsy, often influenced by factors such as age, adherence to treatment, and seizure type^40^. However, recent research by^41^ from northern Ethiopia reported that 60.3% (95% CI: 55.6–65.0%) of study participants achieved seizure remission for at least one year, further highlighting the variability in remission rates across different populations. This indictes that our remission rate does not overlap with that of the Northern Ethiopia study, suggesting a statistically significant difference. Similarly, a study involving a large cohort of Scottish patients reported a seizure remission rate of 63.7% for at least one year, suggesting that a significant proportion of patients had not achieved remission^42^. Furthermore, the remission rate observed in this thesis is notably lower than that of a study conducted in China, which reported a remission rate of 80%^43^. Additionally, the remission rate in this study is lower than previous research on diagnosed epilepsy, which has indicated remission rates slightly above 50%^40^. These discrepancies highlight the variability in remission rates across different populations and underscore the need for further investigation into the factors influencing these outcomes.

This study demonstrated that patients with a high number of pre-treatment seizures were significantly associated with poor seizure remission, as evidenced by prolonged time to remission. Similar findings have been reported in several observational studies in epilepsy, indicating that a higher frequency of seizures prior to treatment negatively correlates with time to remission, thereby extending the duration until remission is achieved^40,44^. The underlying rationale for this association is based on the theory that prolonged and frequent seizures may lead to neuronal damage and the potential development of new epileptic foci^3^. Additionally, a high number of pre-treatment seizures may reflect the intrinsic severity of the epilepsy, which is often associated with a poorer response to antiepileptic drug therapy^45^. This suggests that early recognition of seizures and prompt initiation of appropriate treatment are critical to prevent not only physical injury but also cognitive impairment and psychosocial stigma associated with uncontrolled epilepsy^46^. However, it is noteworthy that some studies have reported no significant association between seizure remission and the number of pre-treatment seizures^47^. These discrepancies may arise from differences in study populations, definitions of remission, and treatment protocols, highlighting the complexity of epilepsy management and the need for individualized treatment approaches.

Patients aged between 25 and 44 years experienced significantly prolonged remission times compared to the 15–24 age group. This observation aligns with previous studies suggesting that older patients tend to have more favourable remission rates^48,49^. For instance^48^, found that older age was associated with a higher likelihood of achieving seizure freedom, potentially due to several factors, including better treatment adherence and the natural progression of the disease. Conversely, several hospital-based cohorts have failed to demonstrate a significant association between age and time to remission^44,50,51^. These studies suggest that while age may play a role in treatment outcomes, it is not a definitive predictor of remission for all patients. Additionally^51^, reported no significant effect of age on treatment outcomes, indicating that individual patient factors, such as seizure types, comorbidities, and treatment regimens, may have a more substantial impact on remission than age alone.

Generalized epilepsy was found to be the predominant type of epilepsy in our study, accounting for 82.8% of cases. This finding contrasts with an epidemiological study conducted in Egypt, which reported a prevalence of generalized epilepsy at 47.6%^52^. Several factors may contribute to these discrepancies, including etiological and ethnic differences among populations. Additionally, the high proportion of generalized epilepsy in developing countries, particularly in Africa, may be influenced by systemic issues such as the limited availability of advanced diagnostic tests and variations in the training of neurologists in interpreting electroencephalograms (EEGs). This situation can lead to misclassification of seizure types, further complicating accurate diagnosis and treatment^42^.

Our study found that focal seizures (FCS) serve as a negative predictor for remission, with patients experiencing significantly longer time to seizure freedom compared to generalized tonic-clonic seizures (GCTS). This finding contrasts with the statement in the previous report that focal epilepsy often has better remission rates^5^. The discrepancy may arise from differences in study populations, etiologies, and healthcare contexts. For example, focal epilepsies in our cohort may be more refractory, less responsive to available treatments, or more heterogeneous in etiology, influencing prolonged remission times. Our results align with previous study reporting poorer remission outcomes for focal epilepsy^40^, while others have found no significant association or better outcomes^53–55^. These inconsistencies highlight the complexity of epilepsy prognosis, where seizure type interacts with other factors such as treatment adherence, comorbidities, and demographic characteristics to influence remission.

Good adherence to antiepileptic therapy is a significant predictor of remission in patients with epilepsy. This finding is consistent with several studies that highlight the critical role of medication adherence in managing seizure control. For instance, a systematic review by^56^ emphasizes that non-adherence to AEDs is a major contributor to uncontrolled seizures and can significantly impact the overall treatment outcomes in epilepsy patients. In a hospital-based cross-sectional study conducted by^57^ at Mizan Hospital, it was found that low medication adherence was an independent predictor of poor remission. This study further indicated that high adherence to AEDs correlates with a decreased risk of seizure attacks, reinforcing the notion that consistent medication intake is vital for effective epilepsy management.

Notably, the most common reason for non-adherence identified in this study was forgetfulness (43.5%), which aligns with findings from other research that suggest cognitive factors play a crucial role in medication adherence^58^. However, this perspective is not universally accepted. Some studies, such as those by^59–61^, have reported no significant difference in adherence to AEDs between remitted and unremitted groups. These studies suggest that factors other than adherence, such as the duration of epilepsy or the specific characteristics of the seizures, may also influence remission outcomes. Additionally, they highlight that both prolonged and shorter times to remission can occur irrespective of adherence levels, indicating a more complex interplay of variables affecting seizure control.

The selection of lognormal baseline hazard combined with an inverse Gaussian frailty distribution was identified as the best-fitting model based on AIC (lowest AIC = 1686.94), outperforming alternatives like Weibull and log-logistic with gamma or inverse Gaussian frailty. The lognormal distribution assumes the logarithm of survival times follows a normal distribution, providing flexibility to model unimodal hazard functions that rise to a peak and then decline^62^; reflecting clinical patterns of seizure remission observed in epilepsy cohorts. The inverse Gaussian frailty distribution captures cluster-level heterogeneity (θ = 0.454), and unobserved effects (here, patient residence districts) with heavier tails than the gamma frailty, thus accommodating greater variability among patients within the same cluster^16,57^. Conventional models like the Weibull-gamma framework, though widely used in epilepsy research^63^ were less suitable here due to their monotonic hazard assumptions and symmetric frailty distribution, which underestimate variability in clustered data. While computationally complex^64^, this approach advances methodological rigor in epilepsy prognosis for sub-Saharan Africa, addressing gaps in existing literature that often overlook geographic clustering^65^.

The estimated shape parameter of 3.762 for the lognormal baseline hazard indicates a unimodal hazard pattern in seizure remission, where the risk of remission initially rises to a peak before declining^32,66^. Clinically, this reflects that remission probability is low right after treatment starts, increases as patients respond, then decreases for those who remain refractory over time^44^. This dynamic hazard aligns with epilepsy’s biological course, distinguishing early responders from patients with more persistent seizures^21^. Explicitly modeling this pattern improves understanding of time-dependent remission risk and aids in optimizing tailored clinical interventions.

### Limitations of the study

The study has several limitations that should be considered when interpreting the findings. First, the analysis was restricted to a specific set of covariates due to limited documented variables in medical records, potentially omitting other significant prognostic factors for epilepsy remission. Additionally, the inclusion of non-adherent patients, while necessary for reflecting real-world conditions, may have contributed to a lower observed remission rate. The requirement that participants have at least one and a half years of treatment could introduce selection bias, as it excludes those who discontinued treatment early due to financial constraints, adverse effects, or severe epilepsy, possibly underrepresenting individuals with poorer outcomes. Medication adherence was assessed solely through the self-reported MARS scale, which is susceptible to recall and social desirability bias, and the lack of longitudinal adherence data limits insight into how compliance changes over time. Furthermore, incomplete information on prior epilepsy treatment before enrollment may have influenced remission outcomes. Although free medication provision likely supported adherence, other potential adherence-influencing factors were not thoroughly examined. These limitations may affect the generalizability of the results, highlighting the need for future studies to address these biases and incorporate more comprehensive data collection methods.

## Conclusion

The study identified the log-normal-inverse-Gaussian shared frailty model as the most appropriate for analyzing epilepsy remission time. Key factors influencing remission time were patient age, seizure type, pre-treatment seizure number, and treatment adherence. While a significant proportion of patients did achieve remission, the timelines for this outcome varied considerably among individuals. The study recommended personalized treatment plans, regular follow-ups to enhance adherence, and patient education on treatment importance and remission variability.

## Supplementary Information

Below is the link to the electronic supplementary material.

Supplementary Material 1

## Data Availability

All data generated and used in the current study are available from the corresponding author upon reasonable request.

## Abbreviations

AEDs: Anti-epileptic drugs
AFT: Accelerated failure time
AIC: Akaike information criterion
EEG: Electroencephalograph
FCS: Focal seizure
GTCS: Generalized tonic-clonic seizure
ILAE: International league against epilepsy
IQR: Interquartile range
JUMC: Jimma university medical center
NICE: National institute for health and care excellence
Pdf: Probability density function
RCT: Randomized clinical trial
SANAD: Standard versus new antieleptic drug trial
SIGN: Scottish intercollegiate guidelines network
WHO: World Health Organization
