# Supplementary material for: A shared frailty model for assessing time to seizure remission in adults with epilepsy
Source: Sci Rep. 2025 Sep 1;15:32195. doi: 10.1038/s41598-025-17991-2 (PMC12402454; doi:10.1038/s41598-025-17991-2)
Supplement: Supplementary file 1 — Supplementary Material 1 [file 41598_2025_17991_MOESM1_ESM.docx]

**Supplementary Material: Detailed Results of Alternative Multivariable Frailty Models**

This supplementary document presents the detailed multivariable analysis results for the alternative frailty models explored in the study: Weibull-Gamma, Weibull-Inverse Gaussian, Lognormal-Gamma, Log-logistic-Gamma, and Log-logistic-Inverse Gaussian frailty models. These results include coefficients, standard errors, acceleration factors, confidence intervals, and p-values for all covariates included in the analysis.

The models generally show consistent patterns of association compared to the selected lognormal-inverse Gaussian frailty model, supporting the robustness of the main findings reported in the manuscript.

**Table S1: Multivariable Analysis Using Weibull-Gamma Frailty Model**

| Covariates | Categories | Coefficient | S.E. | ϕ | 95% CI | p-value |
| --- | --- | --- | --- | --- | --- | --- |
| Intercept |  | 3.7021 | 0.0571 | 40.65 | [34.41, 46.89] | <0.001* |
| Age (in years) | 15-24 (ref) |  |  | 1.00 |  |  |
|  | 25-44 | 0.1150 | 0.0465 | 1.12 | [1.02, 1.24] | 0.013* |
|  | 45-64 | 0.0550 | 0.0553 | 1.06 | [0.95, 1.18] | 0.31 |
|  | ≥65 | 0.0950 | 0.0680 | 1.10 | [0.96, 1.26] | 0.16 |
| Seizure type | GCTS (ref) |  |  | 1.00 |  |  |
|  | FC | 0.1320 | 0.0515 | 1.14 | [1.03, 1.27] | 0.006* |
| Number of seizures before diagnosis | ≤5 (ref) |  |  | 1.00 |  |  |
|  | >5 | 0.0780 | 0.0365 | 1.08 | [1.01, 1.16] | 0.025* |
| Pre-treatment duration (in months) | ≤12 (ref) |  |  | 1.00 |  |  |
|  | >12 | 0.0400 | 0.0417 | 1.04 | [0.96, 1.13] | 0.34 |
| Adherence | Poor (ref) |  |  | 1.00 |  |  |
|  | Good | -0.1100 | 0.0453 | 0.90 | [0.81, 1.00] | 0.039* |
| Comorbidity | No (ref) |  |  | 1.00 |  |  |
|  | Yes | 0.0800 | 0.0503 | 1.08 | [0.98, 1.21] | 0.13 |

θ = 0.420*, τ = 0.188, ρ = 3.900, AIC = 1711.86

**Table S2: Multivariable Analysis Using Weibull-Inverse Gaussian Frailty Model**

| Covariates | Categories | Coefficient | S.E. | ϕ | 95% CI | p-value |
| --- | --- | --- | --- | --- | --- | --- |
| Intercept |  | 3.6578 | 0.0560 | 38.80 | [33.00, 44.61] | <0.001* |
| Age (in years) | 15-24 (ref) |  |  | 1.00 |  |  |
|  | 25-44 | 0.1105 | 0.0452 | 1.12 | [1.02, 1.23] | 0.015* |
|  | 45-64 | 0.0499 | 0.0547 | 1.05 | [0.94, 1.17] | 0.36 |
|  | ≥65 | 0.0890 | 0.0675 | 1.09 | [0.95, 1.25] | 0.18 |
| Seizure type | GCTS (ref) |  |  | 1.00 |  |  |
|  | FC | 0.1370 | 0.0502 | 1.15 | [1.04, 1.28] | 0.004* |
| Number of seizures before diagnosis | ≤5 (ref) |  |  | 1.00 |  |  |
|  | >5 | 0.0753 | 0.0359 | 1.07 | [1.00, 1.14] | 0.050 |
| Pre-treatment duration (in months) | ≤12 (ref) |  |  | 1.00 |  |  |
|  | >12 | 0.0385 | 0.0409 | 1.04 | [0.96, 1.12] | 0.35 |
| Adherence | Poor (ref) |  |  | 1.00 |  |  |
|  | Good | -0.1130 | 0.0448 | 0.89 | [0.80, 0.99] | 0.030* |
| Comorbidity | No (ref) |  |  | 1.00 |  |  |
|  | Yes | 0.0790 | 0.0495 | 1.08 | [0.98, 1.20] | 0.12 |

θ = 0.412*, τ = 0.190, ρ = 3.875, AIC = 1706.55

**Table S3: Multivariable Analysis Using Lognormal-Gamma Frailty Model**

| Covariates | Categories | Coefficient | S.E. | ϕ | 95% CI | p-value |
| --- | --- | --- | --- | --- | --- | --- |
| Intercept |  | 3.4550 | 0.0515 | 33.00 | [28.00, 38.00] | <0.001* |
| Age (in years) | 15-24 (ref) |  |  | 1.00 |  |  |
|  | 25-44 | 0.1255 | 0.0438 | 1.13 | [1.04, 1.24] | 0.004* |
|  | 45-64 | 0.0600 | 0.0530 | 1.06 | [0.96, 1.18] | 0.28 |
|  | ≥65 | 0.0900 | 0.0640 | 1.09 | [0.97, 1.24] | 0.17 |
| Seizure type | GCTS (ref) |  |  | 1.00 |  |  |
|  | FC | 0.1400 | 0.0485 | 1.15 | [1.04, 1.27] | 0.005* |
| Number of seizures before diagnosis | ≤5 (ref) |  |  | 1.00 |  |  |
|  | >5 | 0.0800 | 0.0340 | 1.08 | [1.01, 1.17] | 0.020* |
| Pre-treatment duration (in months) | ≤12 (ref) |  |  | 1.00 |  |  |
|  | >12 | 0.0450 | 0.0377 | 1.05 | [0.97, 1.14] | 0.24 |
| Adherence | Poor (ref) |  |  | 1.00 |  |  |
|  | Good | -0.1180 | 0.0438 | 0.89 | [0.81, 0.98] | 0.008* |
| Comorbidity | No (ref) |  |  | 1.00 |  |  |
|  | Yes | 0.0780 | 0.0488 | 1.08 | [0.99, 1.19] | 0.11 |

θ = 0.455*, τ = 0.182, ρ = 3.775, AIC = 1695.79

**Table S4: Multivariable Analysis Using Log-logistic-Gamma Frailty Model**

| Covariates | Categories | Coefficient | S.E. | ϕ | 95% CI | p-value |
| --- | --- | --- | --- | --- | --- | --- |
| Intercept |  | 3.3100 | 0.0550 | 28.90 | [24.00, 33.80] | <0.001* |
| Age (in years) | 15-24 (ref) |  |  | 1.00 |  |  |
|  | 25-44 | 0.1270 | 0.0430 | 1.14 | [1.05, 1.26] | 0.006* |
|  | 45-64 | 0.0650 | 0.0515 | 1.07 | [0.96, 1.19] | 0.21 |
|  | ≥65 | 0.1000 | 0.0620 | 1.11 | [0.98, 1.26] | 0.12 |
| Seizure type | GCTS (ref) |  |  | 1.00 |  |  |
|  | FC | 0.1480 | 0.0465 | 1.16 | [1.07, 1.29] | 0.003* |
| Number of seizures before diagnosis | ≤5 (ref) |  |  | 1.00 |  |  |
|  | >5 | 0.0880 | 0.0340 | 1.09 | [1.02, 1.17] | 0.009* |
| Pre-treatment duration (in months) | ≤12 (ref) |  |  | 1.00 |  |  |
|  | >12 | 0.0420 | 0.0373 | 1.04 | [0.97, 1.13] | 0.28 |
| Adherence | Poor (ref) |  |  | 1.00 |  |  |
|  | Good | -0.1220 | 0.0433 | 0.88 | [0.80, 0.97] | 0.006* |
| Comorbidity | No (ref) |  |  | 1.00 |  |  |
|  | Yes | 0.0795 | 0.0475 | 1.08 | [0.99, 1.19] | 0.10 |

θ = 0.460*, τ = 0.180, ρ = 3.700, AIC = 1700.53

**Table S5: Multivariable Analysis Using Log-logistic-Inverse Gaussian Frailty Model**

| Covariates | Categories | Coefficient | S.E. | ϕ | 95% CI | p-value |
| --- | --- | --- | --- | --- | --- | --- |
| Intercept |  | 3.2894 | 0.0537 | 27.85 | [22.53, 33.27] | <0.001* |
| Age (in years) | 15-24 (ref) |  |  | 1.00 |  |  |
|  | 25-44 | 0.1301 | 0.0440 | 1.14 | [1.03, 1.26] | 0.009* |
|  | 45-64 | 0.0603 | 0.0527 | 1.06 | [0.96, 1.18] | 0.25 |
|  | ≥65 | 0.1050 | 0.0631 | 1.11 | [0.98, 1.26] | 0.10 |
| Seizure type | GCTS (ref) |  |  | 1.00 |  |  |
|  | FC | 0.1502 | 0.0475 | 1.16 | [1.05, 1.29] | 0.002* |
| Number of seizures before diagnosis | ≤5 (ref) |  |  | 1.00 |  |  |
|  | >5 | 0.0854 | 0.0338 | 1.09 | [1.02, 1.17] | 0.011* |
| Pre-treatment duration (in months) | ≤12 (ref) |  |  | 1.00 |  |  |
|  | >12 | 0.0408 | 0.0371 | 1.04 | [0.97, 1.12] | 0.27 |
| Adherence | Poor (ref) |  |  | 1.00 |  |  |
|  | Good | -0.1205 | 0.0430 | 0.88 | [0.80, 0.97] | 0.007* |
| Comorbidity | No (ref) |  |  | 1.00 |  |  |
|  | Yes | 0.0810 | 0.0480 | 1.08 | [0.99, 1.20] | 0.10 |

θ = 0.448*, τ = 0.181, ρ = 3.695, AIC = 1699.15

**Notes:**

*p < 0.05 was statistically significant

θ = Variance of the random effect

τ = Kendall’s tau (dependence measure)

ρ = shape parameter

ϕ = Acceleration factor

S.E. = Standard error

ref = Reference category
